# Speech Stream Composition Affects Statistical Learning: Behavioral and Neural Evidence

**DOI:** 10.3390/brainsci15020198

**Published:** 2025-02-14

**Authors:** Ana Paula Soares, Dario Paiva, Alberto Lema, Diana R. Pereira, Ana Cláudia Rodrigues, Helena Mendes Oliveira

**Affiliations:** 1 Human Cognition Lab, CIPsi, School of Psychology, University of Minho, 4710-057 Braga, Portugalholiveira@psi.uminho.pt (H.M.O.); 2 Psychological Neuroscience Lab, CIPsi, School of Psychology, University of Minho, 4710-057 Braga, Portugal

**Keywords:** statistical learning, implicit learning, explicit learning, entropy, artificial languages

## Abstract

Statistical learning (SL), the ability to extract patterns from the environment, has been assumed to play a central role in whole cognition, particularly in language acquisition. Evidence has been gathered, however, from behavioral experiments relying on simplified artificial languages, raising doubts on the generalizability of these results to natural contexts. Here, we tested if SL is affected by the composition of the speech streams by expositing participants to auditory streams containing either four nonsense words presenting a transitional probability (TP) of 1 (unmixed high-TP condition), four nonsense words presenting TPs of 0.33 (unmixed low-TP condition) or two nonsense words presenting a TP of 1, and two of a TP of 0.33 (mixed condition); first under incidental (implicit), and, subsequently, under intentional (explicit) conditions to further ascertain how prior knowledge modulates the results. Electrophysiological and behavioral data were collected from the familiarization and test phases of each of the SL tasks. Behavior results revealed reliable signs of SL for all the streams, even though differences across stream conditions failed to reach significance. The neural results revealed, however, facilitative processing of the mixed over the unmixed low-TP and the unmixed high-TP conditions in the N400 and P200 components, suggesting that moderate levels of entropy boost SL.

## 1. Introduction

Statistical learning (SL), the ability to extract patterns from the sensory environment even without intention or awareness, has been assumed to play a central role in whole cognition, particularly in the learning of the rule-governed aspects of language. The first evidence of this comes from a seminal study by Saffran et al. [1], which demonstrated that eight-month-old infants, after being exposed for just two minutes to a continuous speech stream made of the repetition of three-syllable nonsense words (e.g., “gikoba”, “tokibu”, “tipolu”, “gopila”) presented without pauses between each other and with no repetition of the same “word” in a row (e.g., “gikobatokibutipolugopilatokibu”), were able to detect co-occurrences between adjacent syllables—a statistic known as transitional probability (TP)—to extract word-like units from the stream (e.g., “gikoba”). Of note, in that artificial language, the probability of a syllable such as “ko” to be followed by “gi”, or a syllable such as “ba” to follow “ko” is highly likely, whereas the probability of a syllable such as “go” to succeed “ba” is less likely once each “word” could only be followed by another “word” in the stream with the same level of probability. Once TPs were the only cue available to assist “word” segmentation, the results obtained from this seminal study suggested that babies use the statistical regularities embedded in speech to discover “word” boundaries.

Since then, many other works have shown that SL mechanisms are also present in other levels of language acquisition, such as word-referent associations (e.g., [2,3,4]), grammatical categorization (e.g., [5,6]), the establishment of long-distance dependencies (e.g., [7,8,9,10]), and the development of literacy skills (e.g., [11,12,13,14]). However, as Siegelman recently pointed out [15], even if all these studies provide evidence that SL is a powerful mechanism that enables individuals to acquire language so quickly and effortlessly, and the fact that numerous works have shown that humans possess remarkable abilities to detect regularities in the environment and that language is a system full of regularities, this does not necessarily mean that SL mechanisms are used and play a fundamental role in language acquisition.

Alternative approaches claim that SL mechanisms cannot fully account for the processes underlying language acquisition in “real” contexts, as these conclusions were drawn from laboratory studies using oversimplified artificial languages that do not mimic the complexity of natural languages (see [16] and [17] for discussion). Indeed, the vast majority of the SL studies conducted so far have exposed participants not only to a small number of nonsense words (typically four or six), each presented an equal number of times to control for word frequency effects, but also “words” with TPs of 1, meaning that each syllable occurs exclusively in a specific “word” and always in the same syllable position (e.g., [1,18,19,20,21,22]). However, as Soares et al. recently emphasized [23], in natural languages, words show a much more diversified pattern both in terms of syllable length and syllable composition and also in the number of times each word occurs in the language, showing a Zipfian distribution (see [24,25] and also [26,27,28] for evidence in the European Portuguese language).

All these aspects considerably change the proprieties of the input to which participants are exposed in natural vs. artificial languages, casting doubts on the generalizability of the results obtained from lab experiments to “real” contexts (see [29] and also [30] for reviews). Furthermore, these studies often assume that participants are tabula rasae, devoid of any prior knowledge of the language to which they are exposed (see [31,32]). This assumption also fails to reflect how language acquisition occurs in natural contexts, as it is well known that individuals use prior linguistic knowledge to support the acquisition of the new one through mechanisms such as anchoring and/or bootstrapping (e.g., see [33] for details).

To address these issues, recent SL studies have attempted to analyze how SL operates under a broader range of conditions (e.g., [19,20,34,35,36,37,38]). Of particular interest for this paper are the studies conducted by Soares et al. ([23,39,40,41,42]), involving both adult and child participants, with and without developmental language disorder (DLD; previously referred to as specific language impairment [SLI]—see [43] and [44] for details). These studies used the triplet-embedded paradigm introduced by Saffran et al. [1], from which behavioral and electrophysiological (EEG) data were collected during the familiarization phase, allowing the authors to study the processes underlying SL and not only its results. Behavioral data were collected after the exposure phase through the use of the two-alternative forced-choice (2-AFC) task, in which participants were asked to decide which stimuli in a pair—a three-syllable nonsense word presented during familiarization and a foil made of the same syllables in an order not presented before—“sounded more familiar” as in the majority of SL studies (see [18,37,45]).

Specifically, in these studies, Soares and colleagues ([23,39,40,41,42]) exposed participants to speech streams made of the repetition of eight three-syllable nonsense words. Four of these “words” presented TPs of 1, as in Saffran et al.’s [1] study (referred to as *easy* or *high-TP words*), and four presented TPs of 0.33 (referred to as *hard* or *low-TP words*). Note that the syllables in the low-TP words also appeared in other “words” embedded in the stream to closely mimic what occurs in natural languages, where the same syllable (e.g., “cur”) can appear in different words at different syllable positions such as in “*cur*.va.ture”, “in.*cur*.sion” or “re.oc.*cur*”. Moreover, the same participants performed the task, first under implicit conditions (i.e., without any instructions about the nonsense words embedded in the stream as typically observed in SL studies) and, afterwards, under explicit conditions (i.e., with the previous knowledge of the nonsense words embedded in the stream) to further analyze the role that explicit knowledge plays on SL.

The results obtained with adult participants ([23,39,45]) showed that although the overall 2-AFC performance exceeded the chance level in both SL tasks (around 60%)—children’s performance ([39,40,41,42]) was not significantly different from chance as observed in other studies (e.g., [46,47,48]—see also [49] for similar findings in the context of the artificial grammar learning [AGL] task)—it was nevertheless substantially below the levels observed in other SL studies. For example, accuracy rates averaged 74% in [50], 75% in [19], and 89% in [18]. The use of a higher number of “words” than used in previous SL studies (i.e., eight vs. four/six—see [18,19,46]) that were repeated fewer times (60 instead of 100 or more), along with the use of different types of “words” (high- and low-TP words) in the same stream, were pointed out by the authors [23] as potential explanations for the results. It is also important to note that although the 2-AFC differences between high-and low-TP words failed to reach statistical significance in the SL tasks performed under implicit and explicit conditions, in subsequent studies the authors found reliable signs of SL in the 2-AFC task performed under implicit conditions but, surprisingly, only for the low-TP words.

To account for this unexpected result, Soares et al. [45] called attention to an inevitable consequence that the manipulation of the TPs in their studies entailed. Because high-TP words were made of unique syllables that occurred only in specific “words”, conversely to low-TP words whose syllables appeared in different “words” in different syllable positions (first, second, and third), the syllables of the high-TP words occurred three times less frequently than the syllables of the low-TP words. Thus, even though high- and low-TP words appeared exactly the same number of times during exposure to account for word frequency effects in processing, the fact that low-TP words entail syllables that occurred more often might have led participants to choose the stimuli that contained syllables that had occurred more frequently in the 2-AFC post-learning task. The shift from a TP-based strategy to a syllable-frequency-based strategy as suggested by these results is also in line with recent studies showing that the level of entropy—a term originally rooted in thermodynamics, referring to the degree of disorder or randomness within a system, and then adopted in disciplines such as information theory to represent the level of uncertainty or unpredictability in a dataset [51]—interferes with the ease with which TPs/word-like units were extracted from the input (e.g., [34,36,52,53,54]).

Indeed, the concept was recently applied to SL research by Siegelman et al. [54] as an index of stream learnability operationalized as the predictability of a given element (e.g., syllable) based on the probability distribution of all the elements in the stream based on Markov’s formula—see also [36] for another implementation of entropy in SL research using Shannon’s formula. Entropy is thus a global statistic that computes distributional information of the input as a whole and that, together with TPs (a local statistic), has been assumed to affect SL processes and their results (see [29]). Previous studies suggest that the lower the level of entropy, the higher the learning rates (e.g., [36,52,53,54]). Thus, it is possible that the higher level of entropy of the streams used by Soares et al. ([23,39,40,45]), due to both the use of a higher number of “words” than used in previous works and the use of “words” presenting different levels of predictability, might made the stream more complex to process and “words” harder to extract, hence justifying the lower rates of SL observed. Yet, it is also important to point out that there are also other studies suggesting that moderate levels of entropy facilitate SL (see [55,56,57,58,59]) as it provides the “right” level of variability to support pattern detection in line with the Goldilocks principle—SL is optimized in streams that are neither too predictable nor too unpredictable.

Still, the EEG data collected during the familiarization phase in Soares et al. ([23,39,45]) studies with adult participants showed modulations in the N100 and, particularly, in the N400 ERP components, considered the signatures of SL in the brain (e.g., [18,21,22,50]). Specifically, the results revealed enhancements in the N100 component, suggesting an increased sensitivity to the regularities embedded in the input as exposure to the speech stream unfolded (e.g., [60]). Importantly, in the N400 component, results showed larger amplitudes for the high- than for the low-TP words regardless of the condition (implicit vs. explicit) under which the SL task was performed, suggesting facilitated access to high-TP representations in the brain and/or more successful integration of high-TP representations into higher-order language structures, as expected to observe at the behavioral level. As we have been claiming, the inconsistency between the behavioral and EEG results might indicate that the EEG data obtained from the familiarization phase and the behavioral data obtained from the test phase might tap into different processes and mechanisms. Indeed, besides being a post-learning task assessing SL only after exposure, it is also important to highlight that in the 2-AFC task, participants are asked to make explicit judgments about regularities that are expected to be acquired implicitly (i.e., without intention and awareness), which might create not only a mismatch between the “mode of learning” and the “mode of assessing”, but also to allow other meta-cognitive factors to affect the results (for a discussion see [18,23], and [61]). Nonetheless, it is also important to consider that the disparity in the results observed by Soares et al. ([23,39,45]) could also stem from the complexity (entropy) of the streams used, even though understanding which levels of entropy enhance SL processes without overwhelming the cognitive system, as well as the factors that may influence them (e.g., prior knowledge), are crucial questions that remain largely overlooked in SL research.

This study was designed to shed light on how the composition of the streams presented to participants both under implicit (without previous knowledge of the “words” embedded in the stream) and explicit (with previous knowledge of the “words” embedded in the stream) conditions could impact SL processes and their results by collecting behavioral (2-AFC) and neural (EEG) data. To that purpose, three types of auditory streams were constructed. Each stream contained four three-syllable nonsense words drawn from the study of Soares et al. [23]. Two of those streams were homogenous (*unmixed streams*), in the sense that they contained only one type of “words”, either high-TP (1) or low-TP (0.33) words. The other type was heterogeneous (*mixed streams*), in the sense that half of the three-syllable nonsense words were high-TP (1) and the other half low-TP words (0.33), as in the previous work of Soares et al. ([23,39,40,45]), but using a lower number of three-syllable nonsense words (four) to approach closely previous studies (e.g., [1,18,19,50]). The rationale behind this proposal was that if the number of words affected SL, as we expected, then we should observe higher levels of 2-AFC performance here than in the previous Soares et al. ([23,39,45]) works. Once homogeneous streams with low-TP words present higher levels of entropy (0.48) than streams with high-TP words (0.16), as computed from Markov’s formula, 2-AFC performance was also expected to be better for the unmixed high-TP than for the unmixed low-TP stream condition. These values were computed through the use of Markov’s formula, taking the TPs of all possible transitions (syllable pairs) into account. Note that low-TP words increase the level of entropy in the stream because they increase the level of randomness or uncertainty between syllable transitions. Importantly, if moderate levels of entropy facilitate SL, as claimed by the Goldilocks principle, performance should be better in the mixed condition (0.26 of entropy according to Markov’s formula) than both in the unmixed low-TP and unmixed high-TP conditions, particularly in the SL tasks performed under implicit conditions, once participants cannot rely on prior knowledge to support SL.

Furthermore, if the presence of high- and low-TP words within the same stream encourages the use of a syllable frequency-based strategy rather than a syllable TP-based strategy, as suggested by Soares et al. ([39,45]), recognition rates would be expected to be higher for low-TP words than for high-TP words in the mixed stream condition. However, it is also plausible that with less complex streams, behavioral and neural results may converge, leading to a better 2-AFC performance for high-TP than low-TP words, consistent with the neural-level findings reported by Soares et al. ([23,39,45]). In the same vein, if the streams’ composition affected SL processes, modulations in the N100 and N400 components were expected to be observed across conditions. Specifically, enhancements in the N100 and N400 components, taken as the neural signatures of SL in the brain, should be observed in the mixed high-TP condition relative to the other stream conditions. Moreover, in the mixed condition, it is also possible that high-TP words produce this kind of effect, particularly under explicit conditions, as observed in previous works.

To the best of our knowledge, this is the first study to simultaneously investigate behavioral and neural (EEG) correlates of auditory SL in speech streams made of low- or high-TP words presented in either unmixed or mixed conditions. Unlike previous studies that manipulated the rate of presentation of visual (shapes) information ([54]) or the frequency with which each “word” was presented during exposure ([36]), we adopted a novel approach by directly varying TPs within speech streams. This design closely approximates the complexity of natural linguistic environments, offering new insights into how SL mechanisms operate in more ecologically valid contexts. Additionally, our work uniquely addresses the role of prior knowledge in extracting regularities from streams of varying complexity, an issue largely overlooked in previous research. By integrating behavioral and neural data, this work can not only deepen our understanding of how the brain processes complex information, advancing theoretical models of SL, but also provides critical insights into the role that SL mechanism might effectively play in language acquisition—a hotly debated issue in current SL literature ([16,17]). These findings also hold promise for informing contemporary artificial intelligence (AI) techniques by refining algorithms designed to emulate human-like language learning and enhancing practical AI applications.

## 2. Materials and Methods

### 2.1. Participants

Fifty-six undergraduate students (*M*_age_ = 21.4; *SD*_age_ = 4.0; 47 women) from the University of Minho participated in the study. The sample size was estimated based on previous studies. A similar sample size was also obtained from power analysis conducted using G*Power [62] to achieve 95% power for detecting a medium effect at a significance criterion of α = 0.05 (1 − β = 0.95; α = 0.05) for an effect size of (*U*) = 0.40 (η_p_^2^~0.082). All participants were native speakers of European Portuguese, with normal hearing and no reported history of learning or language disabilities and/or neurological problems. All were right-handed, as assessed by the Portuguese adaptation of the Edinburgh Handedness Inventory ([63,64]). Written informed consent was obtained from each participant. The local Ethics Committee approved the study.

### 2.2. Stimuli

The nonsense words were taken from Soares et al. ([23]). They were made of 32 unique Portuguese syllables recorded by a native speaker of European Portuguese with a duration of 300 ms each. The syllables were evenly distributed across two syllabaries (syllabary A and B—see Table 1) used either in the implicit or explicit versions of the SL tasks (counterbalanced across participants) to avoid interference. In each syllabary, the 16 syllables were concatenated into triplets, presenting either TPs of 1 (high-TP words) or 0.33 (low-TP words). The high-TP words entailed syllables that only occurred in each of those words, such as “tucida” from syllabary A and “todidu” from syllabary B; while low-TP words entailed syllables that occurred in several words at different (initial, medial, and final) syllable positions, such as the syllable “do” in the nonsense words “dotage”, “tidomi”, and “migedo” from syllabary A and the syllable “pi” in the nonsense words “pitegu”, “tepime”, and “megupi” from syllabary B (see Table 1). The high-TP words were used to construct the unmixed high-TP streams, whereas the low-TP words were used to construct the unmixed low-TP streams. Two mixed streams (mixed A and mixed B—made of two of the four “words” of the unmixed high-TP condition and two of the four “words” of the unmixed low-TP condition) were also constructed for control. See Table 1 for an illustration of the type of streams constructed.

**Figure 1:**
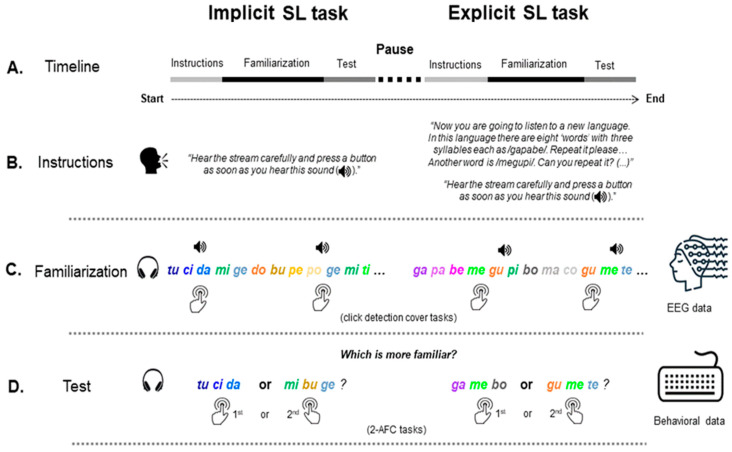
Experimental setup. (Panel **A**) illustrates the timeline of the procedure, beginning with the implicit and subsequently with the explicit SL tasks. Each task comprises three phases: instructions (Panel **B**), familiarization (Panel **C**), and test (Panel **D**). The instructions determine the conditions under which the SL task was performed (implicit vs. explicit). During familiarization, from which EEG data were collected, participants were presented with a continuous auditory stream made of the repetition of the four three-syllable nonsense words (two high- and two low-TP words in the mixed stream condition depicted in the Figure) and instructed to perform the chirp detection task (speaker icon on the Figure). The tasks ended with a test phase (Panel **D**) consisting of a 2-AFC task from which behavioral data were collected.

The nonsense words were concatenated with the Audacity^®^ software (3.7.1. version) with a 50 ms interval between syllables as in previous studies ([39,40,41,42,45]). They were presented in a pseudo-randomized order, such that the same “word” or the same syllable would never appear in a row (i.e., situations such as “tucidatucida” or “tidomimigedo” were not allowed to occur) to avoid confounds (e.g., [1,18,19,20] see also [59]). In each stream, the “words” were presented 60 times in six blocks of 10 repetitions, each lasting 4.2 min (around half a second per block). TPs across “word” boundaries were, therefore, 0.33 for the unmixed high-TP and mixed conditions, and 0.50 for the unmixed low-TP condition. Each stream was also edited to include a superimposed chirp sound (a 0.1 s sawtooth wave sound from 450 to 1450 Hz) in 10% of the syllables to provide participants with a cover task (i.e., a chirp detection task) during exposure, as in previous studies (e.g., [23,39,40,45]). The chirp was included in all “words” counterbalanced across syllable positions to prevent confounds (see Figure 1).

For the 2-AFC tasks, 16 foils were created for each type of stream. The foils were created from the same syllables that made the “words” in each stream and syllabary (see Table 1). The syllables in the foils were used with the same frequency and syllable positions as the syllables in the “words”, though presented in an order not presented before. For example, the syllable “do”, which appeared three times at different syllable positions (initial, medial, final) in three different “words” from the unmixed low-TP condition, also appeared three times at these syllable positions in the foils (e.g., “dogeti”, “midoge”, “timido”—see Table 1). Note, however, that due to stimuli restrictions (i.e., to the number of syllables used to make the four high-TP and the four low-TP words in each syllabary—four vs. twelve), the foils in the unmixed high-TP and the mixed conditions present TPs of 0 as the order of the syllables they entailed was entirely new. However, in the unmixed low-TP condition, the foils present TPs of 0.25, once the number of syllables available and the need to not repeat the same “word” consecutively did not preclude the possibility of a given syllable pair presented in the foils to have occurred during exposure across “word” boundaries (e.g., the syllable pair “geti” presented in the foil “dogeti” could have also appeared when the nonsense word “dotige” was followed by the nonsense “word” “tidomi” in the familiarization phase).

Four lists of materials were created for each type of stream to counterbalance syllables across positions in each syllabary (8 lists per type of stream, 32 lists in total). Participants were randomly assigned to one list of one of the three types of streams (unmixed high-TP, unmixed low-TP, or mixed A or B). A total of 16 participants were assigned to the unmixed high-TP condition, 16 to the unmixed low-TP condition, and 24 to the mixed condition (12 participants to the A and 12 to the B version). Note that when assigned to a given stream condition, participants performed the implicit and the explicit versions of the SL task under the same stream condition (either unmixed high-TP, unmixed low-TP, or mixed), though using stimuli from syllabary A or syllabary B to avoid carry-over effects. The high number of participants assigned to the mixed condition was due to the need to increase the statistical power of the analysis to explore the effects of the type of “word” in the results (note that in this condition, we only use two items per type of “word”).

### 2.3. Procedure

Participants were first presented with the implicit version of the SL task and then with the explicit version of an analogous SL task (see Figure 1). In the implicit version, participants were instructed to pay attention to the auditory stream (a sequence of syllables) presented at 60 dB SPL via binaural headphones. To ensure they paid adequate attention to the stream, a cover task was used during familiarization: participants were asked to detect, as quickly and accurately as possible, a deviant sound (i.e., a chirp sound) that occasionally would appear superimposed over syllables by pressing the spacebar on a computer keyboard. The chirp sound could emerge at any of the three-syllable positions of the “words”, which precluded its use as a cue for stream segmentation. Following exposure, participants were asked to decide, as accurately as possible, which of two auditory stimuli (one “word” and one foil) “sounded more like” the stimuli presented before (i.e., to perform a 2-AFC task). The 2-AFC comprised 16 trials in which each of the four “words” presented during familiarization was paired with the four foils. As in Soares et al. ([39]), this option sought to minimize the number of times each “word” and foil was presented to once as Soares et al. ([45]) have shown that increasing the number of trials by repeating the same stimuli (“words” and foils) several times throughout the 2-AFC task has detrimental effects on SL performance (see ([45]) for details).

In the 2-AFC task, each trial began with the presentation of a fixation point (cross) for 1000 ms, after which the first stimulus (“word” or foil) was presented, followed by the second stimulus. A 500 ms inter-stimulus interval separated the presentation of the stimuli. The next trial began as soon as participants made a response or 10 s had elapsed. The 16 trials were presented in four blocks of four trials each. In each block, the order (first or second) by which the stimuli were presented was controlled, so that in half of the trials, half of the “words” were presented first and in the other half the reverse (counterbalanced across blocks). The trials in each block, as well as the blocks, were randomly presented to the participants.

After a brief interval, participants underwent the explicit version of the SL task. This version followed the same procedure except that, before the new familiarization phase began, the stimuli (i.e., the nonsense words embedded in the stream) were taught. Specifically, participants were told that they would be listening to other “words” from another foreign language. Then, each of the four new “words” was presented auditorily (one by one) and participants were asked to repeat each of them correctly. As in the implicit version of the task, during familiarization, participants were asked to perform the cover task (i.e., press the computer keyboard’s spacebar whenever they heard a chirp sound) to ensure adequate attention to the stream. After familiarization, participants performed another 2-AFC task that mimicked the one used in the implicit version of the SL task.

The procedure took about 60 min to complete per participant. Figure 1 depicts a visual summary of the experimental design adopted in the mixed condition as an example. In all the other conditions, the procedure was exactly the same except for the use of other types of streams (unmixed high-TP and unmixed low-TP streams).

### 2.4. EEG Data Acquisition and Processing

Data collection was performed in an electric-shielded, sound-attenuated room at the facilities of the Psychological Neuroscience Lab (School of Psychology, University of Minho). Participants were seated in a comfortable chair, one meter away from a computer screen. During the familiarization phase, EEG data were recorded with a 64 channels BioSemi Active-Two system (BioSemi, Amsterdam, The Netherlands) according to the international 10–20 system digitized at a sampling rate of 512 Hz and downsampling to 256 Hz. Electrode impedances were kept below 20 kΩ. EEG was re-referenced offline to the algebraic average of mastoids. Data were filtered with a bandpass filter of 0.1–30 Hz (zero-phase-shift Butterworth) plus a notch filter of 50 Hz. Interpolation was performed for electrodes with noise or flat. Independent component analyses (ICA) were performed to remove stereotyped noise (mainly ocular movements and blinks) by subtracting the corresponding components. ERP epochs were time-locked to the nonsense words’ onset, from −300 ms to 1200 ms (baseline correction from −300 to 0 ms). After that, epochs containing artifacts (i.e., with amplitudes exceeding +/−100 µV) were excluded. EEG data processing was conducted with Brain Vision Analyzer, version 2.1.1. (Brain Products, Munich, Germany).

### 2.5. Data Analyses

Behavioral (2-AFC) and EEG data analyses were performed using the IBM-SPSS^®^ software (Version 27.0). All participants showed a click detection performance above 90% during familiarization, except one from the mixed stream condition, which was excluded from the analyses. Therefore, the behavioral analyses considered the data from 55 participants (16 from the unmixed high-TP condition, 16 from the unmixed low-TP condition, and 23 from the mixed condition). For these analyses, the percentage of correct responses was computed for each of the 2-AFC tasks (implicit and explicit) and separately per stream (unmixed high-TP, unmixed low-TP and mixed—mixed A and mixed B collapsed). One-sample *t*-tests against the chance level were conducted on the data of each of the SL tasks and streams to determine whether the performance was significantly different from chance (50%). A repeated measure analysis of variance (ANOVA) using the type of stream (unmixed high-TP, unmixed low-TP, or mixed) as a between-subject factor and the SL task (implicit vs. explicit) as a within-subject factor was also conducted to analyze if 2-AFC performance was significantly different across conditions and groups. For the mixed stream condition, a second ANOVA was conducted to ascertain whether the 2-AFC performance was significantly different across the type of “words” (high- vs. low-TP) and the conditions under which the SL task was performed (implicit vs. explicit).

Individual ERPs were averaged separately per SL task (implicit and explicit), stream condition (unmixed high-TP, unmixed low-TP, or mixed—mixed A and mixed B collapsed), and length of exposure (*half 1*: block#1, block#2, block#3 vs. *half 2*: block#4, block#5, block#6). As in previous studies, we chose to analyze neural data into two different halves to further examine if the brain responses to the different conditions would emerge as a function of the amount of exposure to the input ([23,39,40]). Due to artifact rejection (rejected more than 50% of the trials), six participants were excluded from the unmixed high-TP stream condition in the SL task performed under implicit conditions, and four in the SL task performed under explicit conditions; four participants were excluded from the unmixed low-TP stream condition in the SL task performed under implicit conditions and one in the SL task performed under explicit conditions; and six participants were excluded from the unmixed high-TP stream condition in the SL task performed under implicit and explicit. After artifact rejection, the average accepted trials by condition and group was 83% (178 trials).

Based on previous SL studies (e.g., [18,21,22,23,39,40,50]) and on the general inspection of the data, peak amplitudes were measured for the N100 (60–120 ms) and N400 (350–450 ms) components, taken as the neural signatures of words’ segmentation in the brain. Nonetheless, since a general inspection of the neural results suggested that the positivity observed within the 120–220 ms time window, corresponding to the P200 component, might reveal contrasts of interest, we decided to further examine this component, even though it was not initially considered. Previous SL studies have also reported an enhancement of this component in SL tasks, which has been associated with early perceptual learning of the regularities embedded in the speech streams and attention allocation [65,66,67,68]. To account for the topographical distribution of the abovementioned EEG modulations, peak amplitudes’ values were obtained for the frontocentral regions of interest (ROI; F1, Fz, F2, FC1, FCz, FC2, C1, Cz, and C2) where amplitudes were maximal for the N100, P200, and N400 components.

Repeated measure ANOVAs were conducted on the neural data obtained for the target components in two sets of analyses. In the first, the type of stream (unmixed high-TP, unmixed low-TP, or mixed) was a between-subject factor, and the length of exposure (half 1 vs. half 2) was a within-subject factor. Note that unlike the behavioral analyses and EEG results reported in previous studies ([23,39,40]), here we opted to analyze the SL tasks performed under implicit and explicit conditions separately. This decision was driven by the high number of participants excluded from the EEG analysis—primarily in the unmixed high-TP condition due to artifact rejection. Introducing the SL task condition (implicit vs. explicit) as an additional within-subject factor in the ANOVA would have limited the analysis to only eight participants in the unmixed high-TP condition, significantly reducing statistical power. Thus, for the first EEG analysis, we considered data from 41 participants in the implicit SL task (10 from the unmixed high-TP condition, 12 from the unmixed low-TP condition, and 19 from the mixed condition) and 45 participants in the explicit SL task (12 from the unmixed high-TP condition, 15 from the unmixed low-TP condition, and 18 from the mixed condition). In a second set of ANOVA analyses, focused on the mixed stream condition, we included the SL task (implicit vs. explicit), type of word (high- vs. low-TP), and length of exposure (half 1 vs. half 2) as within-subject factors. This approach was feasible because, in this case, neural data from 17 participants were available for both implicit and explicit SL tasks.

Both for behavioral and ERP data, main and interaction effects that reached statistical or marginal significance levels (*p* < 0.05 or *p* < 0.08, respectively) in comparisons of interest are reported. The Greenhouse–Geisser correction for nonsphericity was used when appropriate. Post hoc tests for multiple comparisons were adjusted with Bonferroni correction. In such cases, the *p*-values reported were the ones obtained after the Bonferroni corrections were automatically applied (i.e., the adjusted *p*-values) by the IBM-SPSS^®^ software (Version 29.0). Measures of effect size (Eta squared, η_p_^2^) and observed power (*pw*) for a single effect are reported in combination with the main effects of the condition. For the behavioral data, the results of one-sample *t*-tests against the chance level were presented first, followed by the results of ANOVAs comparing performance across the three stream conditions, and the ANOVA focused on the mixed stream condition. The EEG results, obtained from the initial ANOVAs conducted for each SL task and the subsequent ANOVA focusing on the mixed stream condition, are reported separately for each targeted ERP component: N100, P200, and N400.

## 3. Results

### 3.1. Behavioral Data

The mean percentages of correct responses obtained for the 2-AFC tasks performed under implicit and explicit conditions per type of stream are presented in Table 2. Standard deviations are presented in parentheses.

The results from the one-sample *t*-tests against chance level showed that participants revealed an above-chance 2-AFC performance in all the conditions, unmixed high-TP: implicit SL task, *t*(15) = 3.96, *p* < 0.001, explicit SL task, *t*(15) = 7.00, *p* < 0.001; unmixed low-TP: implicit SL task, *t*(15) = 2.70, *p* = 0.008, explicit SL task, *t*(15) = 4.20, *p* < 0.001; mixed: implicit SL task, *t*(22) = 3.88, *p* < 0.001; explicit SL task, *t*(22) = 5.39, *p* < 0.001. Moreover, in the mixed condition, the one-sample *t*-tests against chance level showed that performance exceeded the chance levels for both types of “words” in the SL tasks performed under implicit and explicit conditions (see Table 3), high-TP words: implicit SL task, *t*(22) = 3.29, *p* = 0.002, explicit SL task, *t*(22) = 5.54, *p* < 0.001; low-TP words: implicit SL task, *t*(22) = 2.24, *p* = 0.018, explicit SL task, *t*(22) = 3.57, *p* < 0.001.

Additionally, the results from the repeated measures ANOVAs indicated that only the main effect of the SL task reached a statistically significant level, *F*(1, 52) = 12.54, *p* < 0.001, η_p_^2^ = 0.194, *pw* = 0.935. This effect showed that performance was better when the 2-AFC tasks were performed under explicit than implicit learning conditions regardless of the type of stream considered (68.4 vs. 59.8, respectively).

Finally, the results of the ANOVA conducted on the data obtained from the mixed condition showed a main effect of word type, *F*(1, 22) = 5.32, *p* = 0.031, η_p_^2^ = 0.195, *pw* = 0.597, indicating that high-TP words were recognized more accurately than low-TP words (70.4 vs. 61.4, respectively). No other main or interaction effects reached statistical significance.

### 3.2. Electrophysiological Data

#### 3.2.1. N100 Component

**Figure 2:**
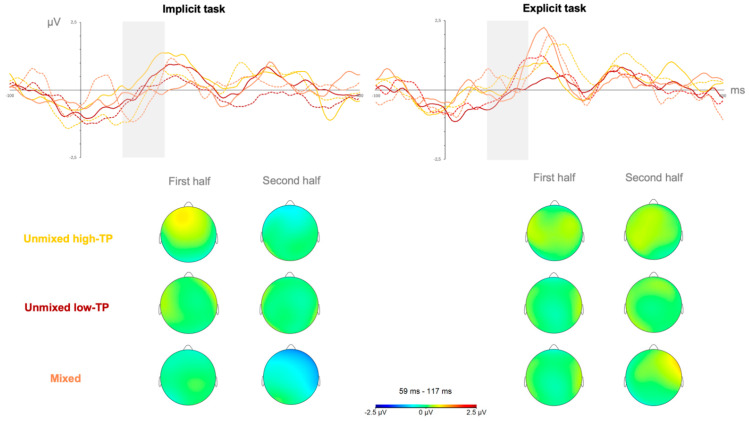
Neural responses in the first and second halves of the SL tasks performed under implicit (**left** panel) and explicit (**right** panel) conditions in the N100 component. Yellow solid line = unmixed high-TP stream, first half; yellow dotted line = unmixed high-TP stream, second half; red solid line = unmixed low-TP stream, first half; red dotted line = unmixed low-TP stream, second half; orange solid line = mixed stream, first half; orange dotted line = mixed stream, second half.

In the first set of analyses, contrasting the three types of streams, the ANOVA revealed that the only effect that reached a statistically significant level was the effect of the length of exposure, *F*(1,38) = 5.234, *p* = 0.028, η_p_^2^ = 0.121, *pw* = 0.606 in the SL task performed under implicit conditions. Figure 2 depicts the neural responses (mean amplitudes’ values and topographical maps) observed in this time window (the gray-shadowed rectangle) for SL tasks performed under implicit (left panel) and explicit (right panel) learning conditions in the first (solid lines) and second (sotted lines) halves for each of the streams (yellow: unmixed high-TP condition; red: unmixed low-TP condition; and orange: mixed condition).

The length of exposure effect observed in the implicit SL tasks in this time window showed a larger amplitude in the second than in the first half of the familiarization phase, as can be inferred from Figure 2 by contrasting, in the gray shadowed rectangle from the left panel, the solid lines with dotted lines in each of the stream conditions.

**Figure 3:**
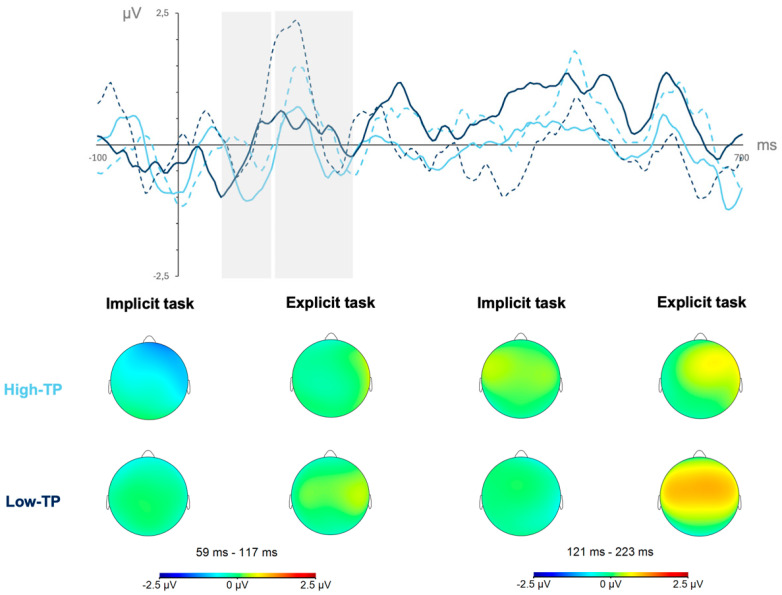
Neural responses from the mixed stream condition in the N100 (first gray shadowed rectangle) and P200 (second gray shadowed rectangle) components for the high-TP (light blue lines) and for the low-TP (dark blue lines) words in the SL tasks performed under implicit (solid lines) and explicit (dotted lines) conditions.

In the second analysis, focused on data from the mixed stream condition, the ANOVA showed that the main effect of word type reached a marginally statistically significant level, *F*(1,16) = 4.111, *p* = 0.060, η_p_^2^ = 0.204, *pw* = 0.478. Figure 3 depicts the neural responses (mean amplitudes’ values and topographical maps) from the mixed stream condition for the high-TP (light blue lines) and the low-TP (dark blue lines) words in the SL tasks performed under implicit (solid lines) and explicit (dotted lines) conditions in the N100 (first gray shadowed rectangle) and P200 (second gray shadowed rectangle) components regardless of the length of exposure to the streams (first and second halves collapsed).

The type of word effect observed in the N100 time window revealed a larger amplitude for the high- than for the low-TP words regardless of the learning conditions (implicit and explicit) under which participants performed the SL tasks, as can be inferred from Figure 3 by contrasting the solid and dotted light blue lines with the solid and dotted dark blue lines in the first gray shadowed rectangle.

#### 3.2.2. P200 Component

**Figure 4:**
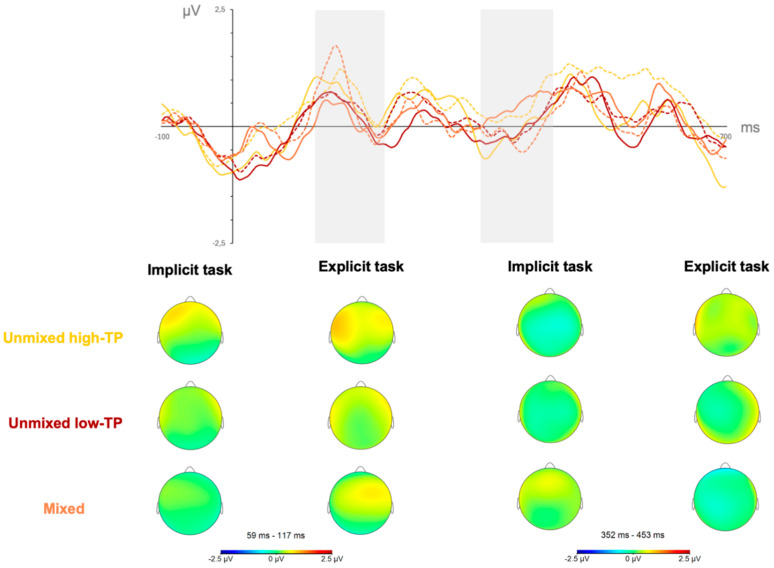
Neural responses in the implicit and explicit SL tasks in the P200 (first gray-shadowed rectangle) and N400 (second gray-shadowed rectangle) components for the SL tasks performed under implicit (solid lines) and explicit (dotted lines) per stream condition: yellow solid line = unmixed high-TP stream, implicit; yellow dotted line = unmixed high-TP stream, explicit; red solid line = unmixed low-TP stream, implicit; red dotted line = unmixed low-TP stream, explicit; orange solid line = mixed stream, implicit; orange dotted line = mixed stream, explicit.

The results from the first ANOVA, comparing the three type of streams, revealed that the main effect of stream reached a statistically significant level, *F*(2,42) = 3.842, *p* = 0.029, η_p_^2^ = 0.155, *pw* = 0.665 in the SL tasks performed under explicit conditions. In the SL tasks performed under implicit conditions the results failed to show any statistically significant main or interaction effect. Figure 4 depicts the neural responses (mean amplitudes’ values and topographical maps) observed in the P200 (first gray-shadowed rectangle) and N400 (second gray-shadowed rectangle) components for the SL tasks performed under implicit (solid lines) and explicit (dotted lines) learning conditions per stream (yellow: unmixed high-TP condition; red: unmixed low-TP condition; and orange: mixed condition) regardless of the length of exposure (first and second halves collapsed).

The stream effect observed in the P200 component showed a larger amplitude in the mixed than in the unmixed low-TP condition (*p* = 0.031), irrespective of whether participants were exposed to the auditory streams under implicit or explicit conditions, as can be inferred by contrasting the orange solid and dotted lines (mixed condition) with the red solid and dotted lines (unmixed low-TP condition), in the first gray shadowed rectangle of Figure 4.

In the second analysis, restricted to the neural data from the mixed stream condition, the ANOVA revealed that the type of SL task main effect reached a marginally statistically significant level, *F*(1,16) = 4.062, *p* = 0.061, η_p_^2^ = 0.202, *pw* = 0.474. The task effect observed in this time window revealed a larger amplitude for the SL tasks performed under explicit than under implicit conditions, as can be inferred by contrasting, in the second gray shadowed rectangle of Figure 3**,** the light and dark blue dotted lines (explicit conditions) with the dark blue solid lines (implicit conditions).

#### 3.2.3. N400 Component

The results from the first ANOVA comparing the three types of streams revealed a significant main effect of type of stream, *F*(2,42) = 4.387, *p* = 0.019, η_p_^2^ = 0.273, *pw* = 0.727 in the SL tasks performed under explicit conditions, similar to the findings observed in the P200 time window. However, in this case, the post hoc comparisons revealed that the difference across stream conditions reached statistical significance when comparing the mixed with the unmixed high-TP stream conditions, with the first exhibiting a larger amplitude than the former (*p* = 0.017), as can be inferred by contrasting the orange solid and dotted lines (mixed condition) with the yellow solid and dotted lines (unmixed high-TP condition) in the second gray shadowed rectangle of Figure 4. The results of the first ANOVA conducted on neural data from the implicit learning conditions, as well as from the second ANOVA conducted on the neural data from the mixed stream condition, did not reveal any statistically significant main or interaction effects.

## 4. Discussion

The present study examined how the composition of the speech streams affected SL processes and their results. We also aimed to ascertain how the prior knowledge of the three-syllable nonsense words embedded in the speech streams modulated these results. To that purpose, three types of speech streams containing either four high-TP words (i.e., three-syllable nonsense words with TP = 1; unmixed high-TP condition), four low-TP words (i.e., three-syllable nonsense words with TP = 0.33; unmixed low-TP condition), or two high-TP and two low-TP words (mixed condition) were presented to participants, first under incidental (implicit), and, subsequently, under intentional (explicit) conditions, as in the previous SL studies of Soares et al. ([23,39,40,41,42]). Neural and behavioral data were collected during each SL task’s familiarization and test phases. To the best of our knowledge, this paper is the first to examine these issues directly with important theoretical and practical implications. The findings can contribute not only to deepening our understanding of how SL mechanisms work in a wide range of situations, approaching closely what might occur in natural languages acquisition; but also to shed light on the type of statistics (local statistics vs. global statistics) participants are sensitive to and use to extract word-like units from the continuous auditory streams, which can be used to inform the advancement of AI techniques and the efficiency of its real-world applications. The results obtained were straightforward and can be summarized as follows: (i) behavioral (2-AFC) signs of SL were observed for the three types of streams and learning conditions, particularly in the SL task performed under explicit conditions, as anticipated; (ii) although 2-AFC performance was numerically higher (and very similar) in the mixed and unmixed high-TP conditions than in the unmixed low-TP condition both in the SL tasks performed under implicit and explicit conditions, differences failed to reach statistical significance across stream conditions; (iii) still, the 2-AFC data obtained from the mixed stream condition revealed a word type effect indicating better recognition rates for the high- than for the low-TP words; (iv) importantly, the neural data showed evidence of stream effects both in the N400 component, foreseen from the outset, and in the additional P200 component showing a larger amplitude in the mixed vs. the unmixed high-TP condition in the former and a larger amplitude in the mixed vs. the unmixed low-TP condition in the latter, suggesting, in both cases, facilitative processing of the mixed over the other stream conditions; (v) additionally, the neural data revealed a length of exposure effect in the N100 component, indexed by a larger amplitude in the second half than in the first half of the SL tasks performed under implicit conditions, regardless of the type of stream, suggesting that participants were able to extract the regularities embedded in the speech streams as exposure unfolded as expected; (vi) a marginally significant word type effect was observed in the N100 component for the mixed stream condition, with high-TP words eliciting larger amplitudes than low-TP words; (vii) although direct comparisons of neural responses between SL tasks were limited to the mixed condition, the separate analyses for the implicit and explicit SL tasks revealed that the stream effects observed in the P200 and N400 components emerged only under explicit conditions, suggesting an indirect SL task effect in the expected direction; and (viii) that a significant (albeit marginally) direct SL task effect was observed in the mixed stream condition, indicated by a larger P200 amplitude in the SL task performed under explicit than implicit conditions, as expected.

These results are interesting and globally provide support for our hypotheses. Indeed, when the number of words embedded in the streams is halved (from eight in the previous work of Soares et al. ([23,39,40,41,42]) to four), an increase in 2-AFC performance was observed, particularly in the SL tasks performed under implicit conditions, as expected. Specifically, a comparison between the results obtained here and those reported by Soares et al. ([39])—these works are more directly comparable since both used 16 trials in the 2-AFC post-learning task—revealed notable differences. When considering overall 2-AFC performance (i.e., regardless of word type), participants improved their performance by 9.5% in the implicit SL tasks (52.8% in Soares et al. [39] vs. 62.2% here) and by 6.1% in the explicit SL tasks (63.5% in Soares et al. [39] vs. 69.6% here). It is also worth noting that when we consider the type of word in the analyses, it is readily apparent that the increase in the 2-AFC performance was more pronounced for the high-TP than for the low-TP words. Indeed, the comparison of the results obtained here in the mixed stream condition with those obtained by Soares et al. [39] revealed that the 2-AFC performance for the high-TP words increased by 18.7% in the implicit SL tasks (47.1% in Soares et al. [39] vs. 65.8% obtained here), and by 13.6% in the explicit SL tasks (61.4% in Soares et al. [39] vs. 75% obtained here). For the low-TP words, however, the comparisons revealed a pretty similar pattern of results across studies with a difference of 0.3% in the SL tasks performed under implicit conditions (58.4% in Soares et al.’s [39] vs. 58.7% obtained here) and a difference of 1.4% in the SL tasks performed under explicit conditions (65.5% in Soares et al. [39] vs. 64.1% obtained here).

In the case of the unmixed streams, the comparisons of the results obtained by Soares et al. [39] for the high-TP words and those obtained here for the unmixed high-TP stream condition revealed an increase of 14.2% in the SL tasks performed under implicit conditions (47.1% in Soares et al. [39] vs. 61.3% obtained here) and an increase of 10.1% in the SL tasks performed under explicit conditions (61.4% in Soares et al. [39] vs. 71.5% obtained here). In the case of unmixed low-TP streams, however, the comparisons revealed a decrease of 2.5% in the SL tasks performed under implicit conditions (58.4% in Soares et al. [39] vs. 55.9% obtained here) and a decrease of 1.4% in the SL tasks performed under explicit conditions (65.5% Soares et al. [39] vs. 64.1% obtained here). It is worth emphasizing that the magnitude of the SL task effect observed in our data is comparable to that reported by Soares et al. [39], with both showing a large effect size. Taken together, these findings indicate that the complexity (entropy) of the auditory streams presented to participants in the triplet-embedded paradigm—indexed both by the number of “words” embedded in it and “word” TPs—strongly impacts “word” recognition, particularly high-TP words both when the SL tasks are performed under implicit and explicit conditions. This also agrees with the view that the representations generated under incidental and intentional learning conditions are not immune to interference, as several authors have been claiming (e.g., [45,69,70,71,72]). Nevertheless, it is also important to note that the relatively low 2-AFC performance observed in the unmixed low-TP condition relative both to the mixed and the unmixed high-TP conditions could arise not only from the fact that it presents the higher level of entropy (0.48)—possibly making it more difficult to extract word-like units—but also from the fact that the foils used in the 2-AFC task present higher TPs (0.25) than the foils used in the unmixed high-TP and stream mixed conditions (TP = 0) due to the reduced number of syllables used in that condition, as mentioned before (see the Stimuli section), which may have made the foils more difficult to discriminate.

Thus, the stream conditions more directly comparable, at least at a behavioral level of analysis, are the unmixed high-TP and the mixed conditions, which failed to show any statistically significant difference even though the 2-AFC performance was 6.3% higher in the mixed condition over the unmixed low-TP condition and 0.9% higher in the mixed condition over the unmixed high-TP condition. The absence of statistically significant differences across stream conditions at the behavioral level suggests that increasing the unpredictability of the speech streams by manipulating “word” TPs did not hinder 2-AFC discriminations, possibly because the levels of entropy introduced in the speech streams were not high enough to disrupt performance, and/or because the 2-AFC post-learning task used to test SL was not sensitive enough to detect such differences, as discussed ahead.

Nevertheless, the EEG data collected during the exposure phase revealed strong stream effects not only in the N400 component, as anticipated from the outset, but also in the P200 component, which was explored additionally. In both cases, the findings suggest facilitative processing of the mixed stream condition over the unmixed low-TP stream condition (in the case of the P200 component) and of the mixed stream condition over the unmixed high-TP stream condition (in the case of the N400 component). These findings crucially revealed that moderate levels of entropy enhanced (rather than hindered) the extraction of word-like units from speech streams, aligning with the Goldilocks principle. This is, to the best of our knowledge, the first study showing how the composition of the speech streams (operationalized in terms of entropy) modulates the neural correlates of SL, hence extending previous results (see [55,56,57,58,59]). By balancing predictability and variability, the mixed streams used in our study appear to have created the optimal conditions to engage attentional mechanisms, allowing for better detection of the regularities embedded in the input and the extraction of word-like units. Predictability may have provided sufficient stability in the input to enable the brain to detect recurring patterns, while the variability introduced by using high- and low-TP words likely stimulated cognitive processes such as prediction, adaptation, and error detection, which are the core of SL. This combination of elements possibly ensured that the mixed streams used in our study were challenging enough to actively engage participants’ learning systems without overloading their cognitive capacities. Moreover, it is also possible that the balance achieved in the mixed streams between predictability and variability may have also optimized the recruitment of both bottom-up and top-down processes in the extraction of input regularities, explaining the large, albeit marginally significant, SL task effect observed in the P200 component for this stream condition. Bottom-up mechanisms, driven by the statistical properties of the input, would have enabled participants to detect transitions and co-occurrences between syllables. At the same time, top-down mechanisms, such as attention to the previously learned “words”, may also have played a crucial role in integrating this information into perceptual (word-like) units. This result, also observed in other studies (e.g., [19,20,23,39,40,41]), and, indirectly, in the other stream conditions under study—note that the stream effects reported in the P200 and N400 components were only observed in SL tasks performed under explicit conditions—suggests a more successful segmentation of the speech streams when “extra” (metalinguistic) information about the to-be-learned regularities were provided, which also agrees with the behavioral data obtained in the current work.

These findings also appear to suggest that results observed in lab experiments using the triplet-embedded paradigm may actually underestimate the human capacity to extract regularities from the speech input—as they rely on oversimplified languages—and that SL mechanisms may play a more fundamental role in language acquisition in a natural context than previously thought. Language is inherently structured but also variable, and this variability can help learners extract meaningful units at different levels of language processing (such as phonemes, syllables, words, and syntactic structures) more efficiently. Indeed, variability might encourage learners to pay more attention to the patterns embedded in the linguistic input, as they have to distinguish between different instances to find regularities, allowing the system to become “fine-tuned” to capture the subtleties of language to which they are exposed. Moreover, language variability compels learners to continuously adjust their predictions about what comes next. For instance, when a learner encounters a word in a novel context, their prediction mechanisms are challenged, requiring them to adapt and refine their understanding based on the new input. This iterative process of prediction, error detection, and adjustment might not only strengthen the human ability to recognize patterns in the linguistic input but also foster the development of more robust and flexible knowledge, enhancing language competence. This might explain why, in cross-situational word learning studies, both children and adults have been shown to learn novel word-object mappings more effectively when exposed to non-uniform (variable) rather than uniform distributions (see [73,74]). SL mechanisms seem thus to be at the heart of language acquisition processes and enable individuals to acquire language so quickly and effortlessly (see [2,3,4,5,6,7,8,9,10,15], see also [23,39,40,41,42]).

Notably, the facilitative effects observed in the mixed stream condition over both the unmixed high-TP and unmixed low-TP stream conditions were evident even if the neural data indicated that participants were sensitive to the regularities embedded in all the speech streams, as reflected by the strong length of exposure effect observed in the N100 component—in line to that reported by Soares et al. [39], although at a later (N400) time window; as well as by the behavioral signs of SL which were observed across all streams conditions. The disparity between behavioral and neural results in SL research is not new (see [18,19,20,23,39,40,41,42,47,48]) and might reflect, as several authors have been claiming, that online (EEG) and offline (2-AFC) SL measures tap into distinct cognitive processes, the first related to how the brain computes statistical regularities embedded in the input, and the second with how people retrieve patterns learned from memory (see [23,37], for a discussion). This also aligns with other works claiming that the 2-AFC task is not suitable for testing SL, as it presents low sensitivity to detect subtle differences as typically used in SL experiments (see [15,45,48]). These findings highlight the importance of using alternative measures to assess SL, particularly those sensitive to the time course of processing, such as EEG, during exposure to the speech streams, as used here and in an increasing number of SL studies ([18,19,20,22,23,33,35,39,40,41,42,47,50,53,67]).

Nevertheless, the 2-AFC results observed in the mixed condition revealed that participants were significantly better at recognizing high-TP than low-TP words both under implicit and explicit conditions. These findings are not in accordance with the 2-AFC results observed previously by Soares et al. ([23,39,45]) but align both with the neural data observed by Soares et al. ([23,39]), who found greater N400 amplitudes for high- than for the low-TP words, and with the large, albeit marginally statistically significant, word type effect observed here for the mixed stream condition in the N100 component. This behavioral result is relevant not only because it is the first converging with neural findings suggesting facilitated processing of high-TP over low-TP words in components like the N100 and N400 (see Soares et al. [23,39]) but also because it rules out the explanation advanced by Soares et al. [45] to account for the better recognition rates observed in their study for the low- than for the high-TP words. Indeed, Soares et al. [45] claimed that because the syllables of the low-TP words were presented three times more often than the syllables of the high-TP words (that entailed unique syllables), this may have led the cognitive system to adopt a syllable-frequency-based strategy rather than a TP-based strategy to recognize the “words” in the 2-AFC post-learning task. The results obtained here do not support this explanation. However, it is important to note that the entropy levels of the mixed streams used in this study—containing two high-TP and two low-TP words—are lower than those used by Soares et al. [45], which included four high-TP and four low-TP words. It is thus possible that the lower complexity of the streams used here had allowed a more efficient extraction of the statistical regularities embedded in the input and the formation of more stable representations of the perceptual units (“words”) in long-term memory, hence avoiding the shift from a TP-based strategy to a syllable-frequency-based strategy that the complexity of the streams used by Soares et al. ([39,45]) seems to have stimulated. In sum, even though the results obtained here are interesting and contribute to existing research by demonstrating that speech streams with moderate levels of entropy create better conditions for SL—likely by striking a balance between predictability and variability to facilitate the extraction of regularities embedded in the speech input—conclusions should be drawn with caution. It is important for future studies to replicate these findings, employing not only a larger number of participants (given the high number of exclusions due to EEG artifact rejection) but also using a within-subject design in the manipulation of the type of stream to minimize the impact that individual differences might have on the results. Nonetheless, these findings are promising and have the potential to open new avenues in SL research. Future studies can further examine the conditions under which entropy might disrupt 2-AFC performance by increasing the number of “words” embedded in the streams or reducing the number of times each “word” is presented to avoid potential ceiling effects. The use of other tasks, such as a target detection post-learning task (see [19] and also [45] for a review), should also be considered. Identifying the “sweet spot” for entropy, as well as exploring the role that prior knowledge plays in determining the “right” level of entropy in different developmental stages, are also crucial questions that future research should address, contributing to achieving a deeper and more comprehensive understanding of the role that SL mechanisms play in “real-world” language acquisition contexts. These insights have the potential to shape the development of cutting-edge AI technologies by refining algorithms that more closely mirror human-like language learning processes. By incorporating principles such as the balance between predictability and variability and adapting to local and global regularities, AI systems can become better equipped to handle complex and dynamic linguistic input in real-world situations, such as human–computer interactions and virtual settings. These advancements could also drive improvements across a wide range of applications, including enhancing the accuracy and adaptability of speech recognition systems, advancing the diagnosis and treatment of language impairments, and improving tools designed to support foreign language learning. Ultimately, bridging the gap between human SL mechanisms and AI algorithms could lead to breakthroughs in how machines process, understand, and interact with language, paving the way for technologies that are more intuitive, adaptable, and effective in meeting the complexities of human communication.

## 5. Conclusions

This work is, to the best of our knowledge, the first providing evidence on how the complexity (entropy) of the speech streams presented to participants in the triplet-embedded paradigm affects SL processes and their results by collecting behavioral (2-AFC) and neural (EEG) data. Although the 2-AFC results failed to reveal significant differences across stream conditions, the neural results crucially indicated facilitative processing of the mixed over the other stream conditions, thus suggesting that SL may play a more fundamental role in language acquisition than previously thought, as the mixed stream condition mimic closely the complexity of natural languages. Although these results should be confirmed with further research, the present work opens new avenues for future SL and AI research.

## Figures and Tables

**Table 1:** Examples of the “words” and foils used per stream condition.

Type of Stream	Syllabary A		Syllabary B	
	“Words”	Foils	“Words”	Foils
unmixed high-TP	tucida	tubago	todidu	tomabe
	bupepo	bucica	cegita	cedico
	modego	mopeda	gapabe	gagidu
	bibaca	bidepo	bomaco	bopata
unmixed low-TP	dotige	dogeti	pitegu	pigute
	tidomi	timido	tepime	temepi
	migedo	midoge	megupi	mepigu
	gemiti	getimi	gumete	guteme
mixed A	migedo	gebado	megupi	gumapi
	gemiti	mogeti	gumete	gagute
	modego	bimigo	gapabe	bomebe
	bibaca	mideca	bomaco	mepaco
mixed B	dotige	docimi	pitegu	tegigu
	tidomi	budoge	tepime	toteme
	tucida	tutipo	todidu	cepidu
	bupepo	tipeda	cegita	pidita

**Table 2:** Mean percentages (and standard deviations) of correct responses for 2-AFC tasks performed under implicit and explicit conditions per type of stream.

	SL Task	
Type of Stream	Implicit	Explicit
Unmixed high-TP	61.3 (11.5)	71.5 (12.3)
Unmixed low-TP	55.9 (8.7)	64.1 (13.4)
Mixed	62.2 (15.1)	69.6 (17.4)

**Table 3:** Mean percentages (and standard deviations) of correct responses for 2-AFC tasks performed under implicit and explicit conditions in the mixed stream condition per type of “word”.

	SL Task	
Type of Word	Implicit	Explicit
High-TP	65.8 (23.0)	75.0 (21.7)
Low-TP	58.7 (18.6)	64.1 (19.0)

## Data Availability

Data from this study can be downloaded at https://osf.io/g8xem/?view_only=250d9e5fc1344022b6818e98bdfd1055 (accessed on 10 February 2025).

## References

[1] Saffran J.R., Aslin R.N., Newport E.L. (1996). Statistical learning by 8-month-old infants. Science.

[2] Breen E., Pomper R., Saffran J. (2019). Phonological learning influences label-object mapping in toddlers. J. Speech Lang. Hear. Res..

[3] Estes K.G., Evans J.L., Alibali M.W., Saffran J.R. (2007). Can infants map meaning to newly segmented words? Statistical segmentation and word learning. Psychol. Sci..

[4] Hay J.F., Pelucchi B., Estes K.G., Saffran J.R. (2011). Linking sounds to meanings: Infant statistical learning in a natural language. Cogn. Psychol..

[5] Mintz T.H. (2003). Frequent frames as a cue for grammatical categories in child directed speech. Cognition.

[6] Monaghan P., Christiansen M.H., Chater N. (2007). The phonological-distributional coherence hypothesis: Cross-linguistic evidence in language acquisition. Cogn. Psychol..

[7] Gómez R., Maye J. (2005). The developmental trajectory of nonadjacent dependency learning. Infancy.

[8] Hsu H., Tomblin J., Christiansen M. (2004). Impaired statistical learning of non-adjacent dependencies in adolescents with specific language impairment. Front. Psychol..

[9] Kidd E. (2012). Implicit statistical learning is directly associated with the acquisition of syntax. Dev. Psychol..

[10] Newport E.L., Aslin R.N. (2004). Learning at a distance I. Statistical learning of non-adjacent dependencies. Cogn. Psychol..

[11] Arciuli J., Simpson I.C. (2012). Statistical learning is related to reading ability in children and adults. Cogn. Sci..

[12] Lages A., Oliveira H.M., Arantes J., Gutiérrez-Domínguez F.J., Soares A.P., Buela-Casal G. (2022). Drawing the links between statistical learning and children’s spoken and written language skills. International Handbook of Clinical Psychology.

[13] Sawi O.M., Rueckl J. (2019). Reading and the neurocognitive bases of statistical learning. Sci. Stud. Read..

[14] Spencer M., Kaschak M.P., Jones J.L., Lonigan C.J. (2015). Statistical learning is related to early literacy-related skills. Read. Writ..

[15] Siegelman N. (2020). Statistical learning abilities and their relation to language. Lang. Linguist. Compass.

[16] Johnson E.K., Rebuschat P., Williams J. (2012). Bootstrapping language: Are infant statisticians up to the job?. Statistical Learning and Language Acquisition.

[17] Yang C.D. (2004). Universal grammar, statistics or both?. Trends Cogn. Sci..

[18] Batterink L.J., Paller K.A. (2017). Online neural monitoring of statistical learning. Cortex.

[19] Batterink L.J., Reber P.J., Paller K.A. (2015). Functional differences between statistical learning with and without explicit training. Learn. Mem..

[20] Batterink L., Reber P.J., Neville H., Paller K.A. (2005). Implicit and explicit contributions to statistical learning. J. Mem. Lang..

[21] Cunillera T., Càmara E., Toro J.M., Marco-Pallares J., Sebastián-Galles N., Ortiz H. (2009). Time course and functional neuroanatomy of speech segmentation in adults. Neuroimage.

[22] Sanders L.D., Ameral V., Sayles K. (2009). Event-related potentials index segmentation of nonsense sounds. Neuropsychologia.

[23] Soares A.P., Gutiérrez-Domínguez F., Vasconcelos M., Oliveira H.M., Tomé D., Jiménez L. (2020). Not all words are equally acquired: Transitional probabilities and instructions affect the electrophysiological correlates of statistical learning. Front. Hum. Neurosci..

[24] Zipf G. (1936). The Psychobiology of Language.

[25] Piantadosi S.T. (2014). Zipf’s word frequency law in natural language: A critical review and future directions. Psychon. Bull. Rev..

[26] Soares A.P., Iriarte A., Almeida J.J., Simões A., Costa A., França P., Machado J., Comesaña M. (2014). Procura-PALavras (P-PAL): Uma nova medida de frequência lexical do português europeu contemporâneo. Psicol. Reflex. Crit..

[27] Soares A.P., Lages A., Silva A., Comesaña M., Sousa I., Pinheiro A.P., Perea M. (2019). Psycholinguistics variables in the visual-word recognition and pronunciation of European Portuguese words: A megastudy approach. Lang. Cogn. Neurosci..

[28] Campos A.D., Oliveira H.M., Soares A.P. (2018). The role of syllables in intermediate-depth stress-timed languages: Masked priming evidence in European Portuguese. Read. Writ..

[29] Thiessen E.D., Kronstein A.T., Hufnagle D.G. (2013). The extraction and integration framework: A two-process account of statistical learning. Psychol. Bull..

[30] Hasson U. (2017). The neurobiology of uncertainty: Implications for statistical learning. Philos. Trans. R. Soc. B.

[31] Stärk K., Kidd E., Frost R.L. (2022). The effect of children’s prior knowledge and language abilities on their statistical learning. Appl. Psycholinguist..

[32] Siegelman N., Bogaerts L., Elazar A., Arciuli J., Frost R. (2018). Linguistic entrenchment: Prior knowledge impacts statistical learning performance. Cognition.

[33] Cunillera T., Laine M., Càmara E., Rodríguez-Fornells A. (2010). Bridging the gap between speech segmentation and word-to-world mappings: Evidence from an audiovisual statistical learning task. J. Mem. Lang..

[34] Bogaerts L., Siegelman N., Frost R. (2016). Splitting the variance of statistical learning performance: A parametric investigation of exposure duration and transitional probabilities. Psychol. Bull..

[35] Gutiérrez-Domínguez F.J., Lages A., Oliveira H.M., Soares A.P., Buela-Casal G. (2022). Neural signature of statistical learning: Proposed signs of typical/atypical language functioning from EEG time-frequency analysis. International Handbook of Clinical Psychology.

[36] Lavi-Rotbain O., Arnon I. (2022). The learnability consequences of Zipfian distributions in language. Cognition.

[37] Siegelman N., Bogaerts L., Frost R. (2017). Measuring individual differences in statistical learning: Current pitfalls and possible solutions. Behav. Res. Methods.

[38] Bogaerts L., Siegelman N., Christiansen M.H., Frost R. (2022). Is there such a thing as a ‘good statistical learner’?. Trends Cogn. Sci..

[39] Soares A.P., Gutiérrez-Domínguez F.J., Lages A., Oliveira H.M., Vasconcelos M., Jiménez L. (2022). Learning Words While Listening to Syllables: Electrophysiological Correlates of Statistical Learning in Children and Adults. Front. Hum. Neurosci..

[40] Soares A.P., Gutiérrez-Domínguez F., Oliveira H.M., Lages A., Guerra N., Pereira A.R., Tomé D., Lousada M. (2022). Explicit instructions do not enhance auditory statistical learning in children with developmental language disorder: Evidence from ERPs. Front. Psychol..

[41] Soares A.P., Lages A., Oliveira H.M., Gutiérrez-Domínguez F.J., Botinis A. (2021). Extracting word-like units when two concurrent regularities collide: Electrophysiological evidence. Proceedings of the 12th International Conference of Experimental Linguistics.

[42] Soares A.P., Lages A., Oliveira H.M., Gutiérrez-Domínguez F.J., Buela-Casal G. (2022). Can explicit instructions enhance auditory statistical learning in children with Developmental Language Disorder?. International Handbook of Clinical Psychology.

[43] Bishop D.V.M., Snowling M.J., Thompson P.A., Greenhalgh T., The CATALISE Consortium (2017). Phase 2 of CATALISE: A multinational and multidisciplinary Delphi consensus study of problems with language development: Terminology. J. Child. Psychol. Psychiatry.

[44] Soares A.P., Lousada M., Ramalho M., Alves R.A., Leite I. (2021). Perturbação do desenvolvimento da linguagem: Terminologia, caracterização e implicações para os processos de alfabetização [Language development disorder: Terminology, characterization and implications for literacy]. Alfabetização Baseada na Ciência.

[45] Soares A.P., França T., Gutiérrez-Domínguez F., Sousa I., Oliveira H.M. (2022). As trials goes by: Effects of 2-AFC item repetition on SL performance for high- and low-TP ‘Words’ under implicit and explicit conditions. Can. J. Exp. Psychol..

[46] Lukács Á., Lukics K.S., Dobó D. (2021). Online Statistical Learning in Developmental Language Disorder. Front. Hum. Neurosci..

[47] Raviv L., Arnon I. (2018). The developmental trajectory of children’s auditory and visual statistical learning abilities: Modality-based differences in the effect of age. Dev. Sci..

[48] van Witteloostuijn M., Lammertink I., Boersma P., Wijnen F., Rispens J. (2019). Assessing visual statistical learning in early-school-aged children: The usefulness of an online reaction time measure. Front. Psychol..

[49] Soares A.P., Silva R., Faria F., Mendes H.O., Jiménez L. (2021). Literacy effects on artificial grammar learning (AGL) with letters and colors: Evidence from preschool and primary school children. Lang. Cogn..

[50] Abla D., Katahira K., Okanoya K. (2008). On-line assessment of statistical learning by event-related potentials. J. Cogn. Neurosci..

[51] Shannon C. (1948). Claude Shannon. Inf. Theory.

[52] Daikoku T., Yumoto M. (2023). Order of statistical learning depends on perceptive uncertainty. Curr. Res. Neurobiol..

[53] Daikoku T., Yatomi Y., Yumoto M. (2017). Statistical learning of an auditory sequence and reorganization of acquired knowledge: A time course of word segmentation and ordering. Neuropsychologia.

[54] Siegelman N., Bogaerts L., Frost R. (2019). What Determines Visual Statistical Learning Performance? Insights From Information Theory. Cogn. Sci..

[55] Aslin R.N., Newport E.L. (2012). Statistical learning: From acquiring specific items to forming general rules. Curr. Dir. Psychol. Sci..

[56] Cubit L.S., Canale R., Handsman R., Kidd C., Bennetto L. (2021). Visual Attention Preference for Intermediate Predictability in Young Children. Child Dev..

[57] Fiser J., Aslin R.N. (2002). Statistical learning of higher-order temporal structure from visual shape sequences. J. Exp. Psychol. Learn. Mem. Cogn..

[58] Kidd C., Piantadosi S.T., Aslin R.N. (2012). The Goldilocks Effect: Human Infants Allocate Attention to Visual Sequences That Are Neither Too Simple Nor Too Complex. PLoS ONE.

[59] Wade S., Kidd C. (2019). The role of prior knowledge and curiosity in learning. Psychon. Bull. Rev..

[60] Heinks-Maldonado T.H., Mathalon D.H., Gray M., Ford J.M. (2005). Fine-tuning of auditory cortex during speech production. Psychophysiology.

[61] Siegelman N., Bogaerts L., Christiansen M.H., Frost R. (2017). Towards a theory of individual differences in statistical learning. Philos. Trans. R. Soc. B.

[62] Faul F., Erdfelder E., Buchner A., Lang A.G. (2009). Statistical power analyses using G*Power 3.1: Tests for correlation and regression analyses. Behav. Res. Methods.

[63] Oldfield R.C. (1971). The assessment and analysis of handedness: The Edinburgh inventory. Neuropsychologia.

[64] Espirito-Santo H., Pires C.F., Garcia I.Q., Daniel F., Silva A.G.D., Fazio R.L. (2017). Preliminary validation of the Portuguese Edinburgh Handedness Inventory in an adult sample. Appl. Neuropsychol. Adult.

[65] Cunillera T., Toro J.M., Sebastián-Gallés N., Rodríguez-Fornells A. (2006). The effects of stress and statistical cues on continuous speech segmentation: An event-related brain potential study. Brain Res..

[66] De Diego Balaguer R., Toro J.M., Rodriguez-Fornells A., Bachoud-Lévi A. (2007). Different neurophysiological mechanisms underlying word and rule extraction from speech. PLoS ONE.

[67] François C., Teixidó M., Takerkart S., Agut T., Bosch L., Rodriguez-Fornells A. (2017). Enhanced Neonatal Brain Responses To Sung Streams Predict Vocabulary Outcomes By Age 18 Months. Sci. Rep..

[68] Pierce L.J., Tague E.C., Nelson C.A. (2021). Maternal stress predicts neural responses during auditory statistical learning in 26-month-old children: An event-related potential study. Cognition.

[69] Eakin D.K., Smith R. (2012). Retroactive interference effects in implicit memory. J. Exp. Psychol. Learn. Mem. Cogn..

[70] Jiménez L., Vaquero J.M.M., Lupiáñez J. (2006). Qualitative differences between implicit and explicit sequence learning. J. Exp. Psychol. Learn. Mem. Cogn..

[71] Lustig C., Hasher L. (2001). Implicit memory is not immune to interference. Psychol. Bull..

[72] Vaquero J.M.M., Lupianez J., Jiménez L. (2019). Asymmetrical effects of control on the expression of implicit sequence learning. Psychol. Res..

[73] Hendrickson A.T., Perfors A. (2019). Cross-situational learning in a Zipfian environment. Cognition.

[74] Yu C., Zhang Y., Slone L.K., Smith L.B. (2021). The infant’s view redefines the problem of referential uncertainty in early word learning. Proc. Natl. Acad. Sci. USA.

